# Social–Emotional Competence for Children with Identified Developmental Concerns: The Impact of Parenting and Executive Function

**DOI:** 10.3390/bs14121232

**Published:** 2024-12-21

**Authors:** Courtney Boise, Lisa L. Knoche

**Affiliations:** 1 Department of Psychology, University of North Florida, Jacksonville, FL 32224, USA; 2 Nebraska Center for Research on Children, Youth, Families and Schools, University of Nebraska-Lincoln, Lincoln, NE 68588, USA; lknoche2@unl.edu

**Keywords:** parenting, parent–child conflict, executive function, social–emotional development, developmental concern

## Abstract

Social–emotional competence and executive function at preschool are critical for children’s school readiness. Unfortunately, young children with the dual risk of low-income status and identified developmental concerns are more likely to have lower social–emotional learning. This study examines (a) bidirectional associations between dyadic parenting behaviors, executive function, and social–emotional competence; and (b) executive function as an explanatory mechanism for the predictive relationship between specific parenting behaviors and children’s social–emotional competence. Data came from 267 parents and children with identified developmental concerns attending publicly funded center-based preschools. Children’s executive function was assessed by teacher-report, while parenting behaviors and children’s social–emotional competence were observationally assessed. Executive function and children’s social–emotional competence were bidirectionally related across the first year of preschool. Cross-lagged panel models demonstrated that executive function was not an explanatory mechanism for the relationship between parenting behaviors and children’s social–emotional competence. However, higher levels of conflict displayed by parents in the fall of the first year of preschool predicted more conflict during the spring of the same year. Higher levels of conflict in the spring then predicted poorer executive function and social–emotional competence during the spring of the child’s second year of preschool.

## 1. Introduction

Social–emotional competence and executive functioning are critical components of young children’s school readiness [1,2], making it critical to examine factors related to the promotion of these competencies in early childhood. Social–emotional development at the end of preschool predicts long-term success in future relationships [3], academic achievement [4,5], and the likelihood a child will graduate from high school [6]. Executive function in preschool predicts academic achievement [7], lower likelihood of psychopathology [8], and bullying [9] in elementary school.

However, preschool-aged children living in families of low income are at increased risk for delayed development of their executive function [10] and social–emotional skills [11]. Children with low social–emotional skills in preschool are at risk of lower academic achievement [12] and social–emotional competence, even into adolescence [13]. Given the importance of supporting positive social–emotional competence, it is important to examine factors that can predict growth in social–emotional skills, including parenting behaviors and children’s executive function.

Research has shown that parenting is related to executive function [14,15] and social–emotional competence [16,17]. However, we do not know if executive function may serve as an explanatory mechanism for the relationship between parenting and social–emotional skills, that is, if executive function mediates the relationship such that parenting behaviors predict executive function, which in turn predicts social–emotional development. Additionally, we do not know if these relationships operate bidirectionally over time. Understanding these developmental processes will allow for better understanding of interventions for school readiness, long-term academic achievement, and social–emotional development for children.

### 1.1. Parenting and Social–Emotional Development

Social–emotional competencies include the ability to engage in social interactions, recognize other’s emotions, respond to social cues appropriately, and be aware of one’s own actions [18,19]. In a longitudinal study of maltreatment, Egeland and colleagues [20] identified several social–emotional skills for preschool-aged children including high agency, persistence, compliance, and affection towards a parent, as well as low avoidance of the parent and negativity. Across the preschool period, children are able to exhibit more complex social–emotional skills as they develop a defined sense of self, which allows children to navigate between desires for autonomy and relatedness. For example, children’s autonomous behaviors may contribute to their ability to be persistent and have high agency and enthusiasm, while the promotion of relatedness and belonging may cause children to display social–emotional skills such as compliance and affection [21].

In a study of preschool children with developmental delays, greater agency (i.e., a child’s ability to take an active interest in making choices when playing with others, invest enthusiasm, and appreciate successes) was related to more positive social interactions with peers [22]. Similarly, Van Berkel and colleagues [23] demonstrated a relationship between compliance (i.e., a child’s ability to listen to and follow parent’s requests) and engaging in prosocial behavior. Comparatively, greater persistence (i.e., a child’s ability to continue to engage in problem solving and attempt solutions during interactions with others) was related to fewer behavior problems during preschool [24]. In contrast, avoidance (i.e., a child’s attempts to withdraw physically and emotionally from others) was associated with poorer communication with parents and less prosocial behavior with peers [25].

Studies have found an association between parenting behaviors and their children’s future social–emotional development, as the parent–child relationship serves as one of the first sources for socialization and the development of internal working models for future relationships [26,27]. Parenting is a complex construct encompassing multiple dimensions including dyadic parenting behaviors such as parental conflict, reciprocity, and cooperation. Parental conflict behaviors (i.e., the ways in which parents engage in arguing with children) may disrupt optimal child functioning. In a high-risk sample of preschool-aged children, greater levels of parental conflict behaviors were related to increases in disruptive child behavior over time [28]. Weaver and colleagues suggest that an important next step for research is the examination of parental conflict behaviors observationally with similar at-risk populations.

On the other hand, reciprocity is related to early social–emotional skills [29]. Reciprocity predicts children’s positive social–emotional competence from early childhood to adolescence [30]. In a study of children diagnosed with autism, greater parental reciprocity predicted less children’s expression of negativity (i.e., the degree to which the child expresses anger, dislike, or hostility) toward the parent [31].

Parents engaging in less conflict [32] and more cooperative behavior with their preschool children [33] predicted positive social–emotional development across both the school and home contexts. Parents who reported higher levels of conflict behaviors with their children were more likely to have children who displayed lower prosocial behavior and greater peer and emotion problems [32]. Further, Deater-Deckard and Petrill [33] found parental cooperative behavior to be associated with better child social–emotional skills. Further study is needed to better understand explanatory mechanisms for this relationship between parenting behaviors and social–emotional development, especially in the context of risk factors like low socioeconomic status and identified developmental concerns.

### 1.2. Parenting and Executive Function

Executive function may serve as one explanatory mechanism linking parenting behaviors and social–emotional development. Executive functions are foundational cognitive skills that allow children to complete goal-directed activities [34]. Jones and colleagues identified four executive functions for early childhood: (a) inhibition, or the ability to override a dominant response; (b) flexibility, or the ability to switch between tasks or decision rules; (c) attentional control, or the ability to maintain focus on a given task; and (d) working memory, or the ability to monitor, maintain, add, and delete information [35]. Senn and colleagues [36] used path analysis to examine the associations between differentiated executive functions for children ranging in age from 2 years 8 months to 6 years old. While research with adults [34] suggests that inhibition is an underlying component of executive function, this was not supported. Instead, both working memory and inhibition made contributions to problem solving. Wiebe and colleagues [37] extended this exploration of differentiated executive function for preschool children by having 3-year-old children complete a battery of executive function tasks that assessed inhibition, shifting, and working memory. A confirmatory factor analysis supported a single latent construct of executive function for children at this age.

Underlying processes for executive functions begin emerging before children turn one year old. At this age, children demonstrate the ability to inhibit behavior in specific circumstances, such as delayed eating [38]. At one year, children are also able to shift between two physical objects, but they are not yet able to shift between internal representations and the physical environment. An underdeveloped anterior attention system may limit children’s ability to perform these more complex executive functions during the first year of life [39]. The anterior attention system, also referred to as the executive attention network, regulates processes in the anterior cingulate cortex, lateral and ventral prefrontal cortex, and the basal ganglia in order to resolve conflicts caused by differing dimensions of target stimuli. The successful resolution of these conflicts by the anterior attention system is essential for children’s executive function. Furthermore, measuring the resolution of these conflicts is the foundation of many direct assessments of children’s executive function [40].

Between age 3 and 4, and then between 4 and 5, children demonstrate significant growth in executive function [41]. This suggests that the preschool years may be an especially salient opportunity for studying children’s executive function. Therefore, it is important to examine factors, like parenting, which may predict executive function and developmental processes to which executive function may contribute during the preschool years.

Research conducted with Head Start and other early childcare programs demonstrates that preschool children’s executive function predicts school readiness [42] and, later, academic achievement [7]. However, Head Start children exposed to chronic stressors and from families of low income show delays in executive function [43]. Therefore, it is important to examine processes that predict executive function to better promote resilience in young children.

Researchers have demonstrated that high-quality parenting behaviors promote the development of executive function, while low-quality parenting is related to increased problems with executive function [16]. However, parent behaviors do not exclusively impact children’s behaviors; children’s behaviors also impact their parents. Therefore, bidirectional models are needed to examine these associations. For example, Blair and colleagues [15] found that there was a bidirectional relationship between the two variables as high parenting quality predicted growth in executive function, while problems with executive function predicted declining parenting quality.

### 1.3. Executive Function and Social–Emotional Development

Additionally, focusing on children’s executive function can help ameliorate the impact of risk on social–emotional skills. Evidence suggests an association between executive function and children’s social–emotional development in the context of perceived risk. In a study of at-risk preschool-aged children, executive function was related to resilience in social–emotional development. At-risk children who had high executive function displayed similar levels of social–emotional skills compared with typically developing peers [44]. Further research is needed to examine this association using observational assessments of children’s social–emotional skills, as previous research has often examined social–emotional development via parent and teacher reports.

This study has three objectives: (1) are parenting behaviors (i.e., reciprocity, conflict, and cooperation) and executive function bidirectionally related for preschool children with identified developmental concerns?; (2) are executive function and social–emotional skills bidirectionally related for preschool children with identified developmental concerns?; and (3) is executive function an explanatory mechanism for the relationship between dyadic parenting behaviors and social–emotional growth for children with identified developmental concerns longitudinally during the preschool years?

**Hypothesis** **1:** *we hypothesize that parenting behaviors will predict children’s executive function at the subsequent time point. Children’s executive function will also be predictive of later dyadic parenting behaviors.*

**Hypothesis** **2:** *we hypothesize that children’s executive function will predict their later social–emotional skills. In turn, children’s social–emotional skills will predict later executive function.*

**Hypothesis** **3:** *we hypothesize that that executive function will mediate the association between dyadic parenting behaviors and children’s social–emotional skills.*

## 2. Materials and Methods

### 2.1. Sample

Participants were 267 parents (87.2% maternal caregivers) and their children who were enrolled in Head Start or other publicly funded preschools in a Midwestern state between 2012 and 2016 (See Table 1 for demographic information). Participants were part of a study examining Getting Ready, a school readiness intervention [45]. Thirteen publicly funded preschool programs across the Midwestern state participated in the Getting Ready study. Children at these programs were involved in part-day center-based services in preschool settings in both urban and rural communities.

#### Attrition

Participants left the study if they withdrew from the preschool program or if their assigned educator left their position. There were no statistically significant differences in key demographic variables or study variables for those participants who remained in the study compared with those who left the study (*p* > 0.05).

### 2.2. Procedures

Upon obtaining consent from the publicly funded preschools and classroom educators, parents were contacted for permission for their children to complete screeners for eligibility in the original study. Treatment group status (treatment versus comparison) was assigned at the teacher level and was controlled for in all analyses for this study, which does not focus on intervention effects. Consent was collected from all educators and parents for all waves of data collection. As part of these consent forms, families gave permission for video-recorded interactions and other data to be used for secondary data analysis by members of the research team.

Assessments were completed at three time points: (1) fall of the child’s first year of preschool, (2) spring of the child’s first year of preschool, and (3) spring of the second year of preschool. Assessments took place at a location convenient to the family including the home, school, or another community location. Parent–child dyads completed a 15 min play interaction using the Three Box Task Assessment [46]. Parents were instructed to play with their child in the same manner that they normally would at home using materials found in three boxes, which contained items to encourage free play. The first box had stencils, markers, and paper; the second box had a cash register, pennies, and clothes for dress up; and the third box had a set of Lego. Parents chose how to divide the 15 min play interaction across the three boxes.

### 2.3. Measures

#### 2.3.1. Parenting Behaviors

Parenting behaviors were observationally assessed using the Parent–Child Interaction System (PARCHISY) [47] via video-recorded parent–child interactions during the Three Box Task Assessment [46] 15 min play task during fall and spring of the child’s first year of preschool, and spring of the child’s second year of preschool. One third of the video-recorded interactions were double coded by separate team members to ensure continued reliability between coders. Intraclass correlations between coders ranged between 0.80 and 0.95 for all coders across the three parenting behavior codes.

Parenting behaviors included (a) reciprocity, or the ability of the parent to engage in turn-taking with the child; (b) conflict, or the degree to which the parent engages in arguing or mutual negative affect with the child; and (c) cooperation, or the degree to which the parent engages in explicit agreement, discussion, and mutual decision-making with the child about how to proceed during play. Each parenting behavior was rated on a 5-point Likert scale ranging from very low = almost no signs of the behavior to very high = the behavior is a predominant part of the entire interaction. Parenting behaviors on PARCHISY have been shown to demonstrate good reliability, with Cronbach’s alphas ranging from 0.80 to 0.97 [48].

#### 2.3.2. Child Executive Function

This predictor variable was measured using teacher report on the Behavior Rating for Inventory of Executive Function-Preschool Version (BRIEF-P) [49]. Following the guidance of Spiegel and colleagues’ factor analysis of the BRIEF-P [50], this study used a general executive function factor that captures basic executive functions. Previous research has demonstrated a Cronbach’s alpha for overall executive function of 0.90, which demonstrates good reliability [51].

#### 2.3.3. Child Social–Emotional Skills

Social–emotional skills were observationally assessed using the Child Behavior Coding scale [52] during the Three Box Assessment Task [46] during the fall and spring of the child’s first year of preschool, and spring of the second year of preschool. Interactions were observationally coded using the Child Behavior Coding scale, which was developed for a previous iteration of Getting Ready. One-third of the video-recorded interactions were double coded by separate team members to ensure continued reliability between coders. Intraclass correlations between coders ranged between 0.80 and 0.94 for all coders across the five social–emotional skill codes.

Codes for social–emotional skills during parent–child interactions were adapted using rating scales created by Egeland and Sroufe [20,53,54]. Five child social–emotional skills during parent–child interactions were assessed on a 5-point Likert scale. Child behaviors include (1) agency, (2) compliance, (3) avoidance of the parent, (4) affection towards the parent, and (5) negativity.

#### 2.3.4. Demographic/Control Variables

Parents completed demographic surveys reporting parent and child race and ethnicity, as well as child gender. Treatment versus control status was assigned at the teacher level at the start of the study. Treatment status was controlled for in all analyses.

### 2.4. Analytic Strategy

All analyses controlled for the treatment group (treatment or comparison). Multiple and logistic regression models were used to test the hypotheses for Research Questions 1 and 2 of this study using SPSS Version 22. Cross-lagged panel models were used to test the hypothesis for Research Question 3 using Mplus [55]. All participants with at least one measurement were included in analyses for Research Question 3 as missing data were handled using full information likelihood, which allowed for use of the full sample (FML) [56].

## 3. Results

### 3.1. Preliminary Analyses

Table 2 presents descriptive statistics for all variables of interest across the three time points for the study. Parent education, home language, and child gender were all considered as potential covariates for the following analyses. However, none of these variables contributed statistically to the subsequent models and were left out for parsimony. Data were examined for outliers before proceeding with analyses. As no outliers were identified, the full dataset was used in all analyses.

#### 3.1.1. Research Question 1

Regression analyses were conducted to examine parenting behaviors predicting executive function across the first year of preschool, controlling for child executive function in the fall (see Table 3). Parents displaying more conflict behaviors during the fall predicted poorer children’s executive function during the spring of the first year of preschool. Reciprocity and cooperation were not related to children’s subsequent executive function.

Regression analyses were conducted to examine executive function predicting parenting behaviors across the first year of preschool, controlling for parenting behaviors in the fall (see Table 4). More teacher-rated concerns regarding executive function during the fall predicted parents displaying less reciprocity during parent–child interactions during the spring of the first year of preschool. There was no relationship between executive function during the fall and conflict or cooperation displayed by parents during the spring of the first year of preschool.

#### 3.1.2. Research Question 2

Regression analyses were conducted to examine executive function predicting children’s social–emotional skills across the first year of preschool, controlling for children’s social–emotional skills in the fall (see Table 5). Fewer concerns regarding children’s executive function in the fall predicted children expressing more affection towards their parents during the spring of the first year of preschool. Executive function in the fall of the first year of preschool was not related to agency, compliance, avoidance, or negativity during the spring of the first year of preschool.

Regression analyses were conducted to examine social–emotional skills predicting executive function across the first year of preschool, controlling for children’s executive function in the fall (see Table 6). Children displaying less agency and more affection toward their parent during the fall predicted greater teacher-rated concerns regarding children’s executive function during the spring of the first year of preschool. Compliance, avoidance, and negativity during the fall of the first year of preschool were not related to executive function during the spring of the first year of preschool.

#### 3.1.3. Research Question 3

Results from Research Questions 1 and 2 were used to inform analyses conducted to answer Research Question 3. As only predictive relations were demonstrated between agency and affection towards the parent and executive function, agency and affection towards the parent were the only social–emotional outcomes examined in these analyses. As parent conflict behaviors were the only parenting variable that predicted later executive function, cross-lagged panel models were only run to examine this parenting behavior.

**Figure 1:**
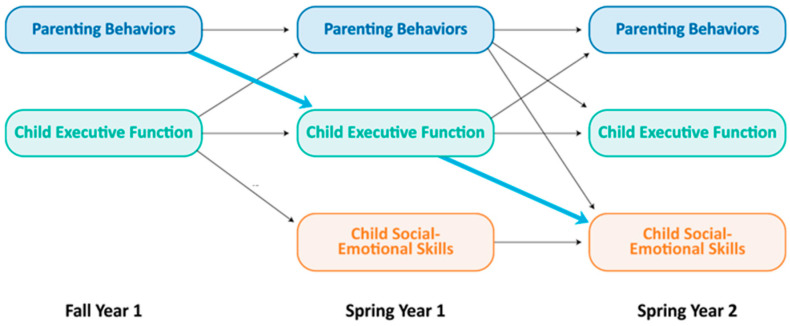
Cross-lagged panel model for Hypothesis 3.

Autoregressive cross-lagged panel models examined executive function as an explanatory mechanism for parent conflict behaviors predicting agency and affection towards the parent (see Figure 1).

**Figure 2:**
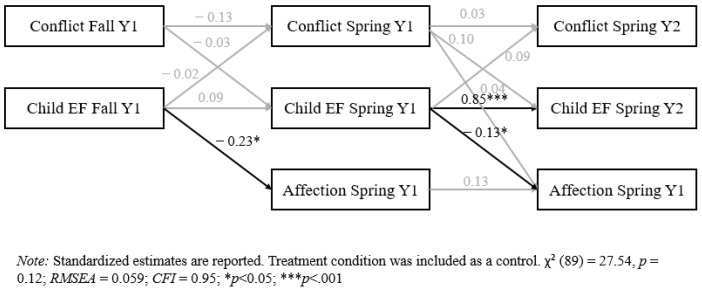
Cross-lagged panel model for conflict, executive function, and child affection towards the parent.

The model examining conflict behaviors, executive function, and child affection towards the parent demonstrated good model fit(see Figure 2). However, there were no significant indirect effects in this cross-lagged panel model. Echoing results from the regression analyses, executive function predicted affection towards the parent at the subsequent time point (fall of the first year to spring of the first year; spring of the first year to spring of the second year).

**Figure 3:**
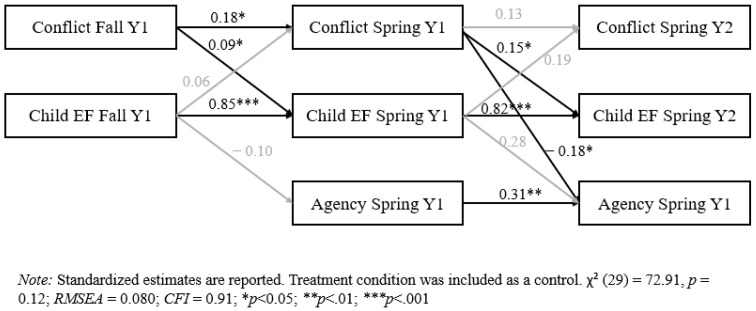
Cross-lagged panel model for conflict, executive function, and child agency.

The model examining conflict behaviors, executive function, and child agency demonstrated good model fit (see Figure 3). Parents displaying more conflict behaviors during the fall of the first year of preschool predicted more conflict behaviors and more problems with child executive function during the spring of the first year of preschool. Parents displaying more conflict behaviors during the spring of the first year of preschool predicted more problems with child executive function and less child agency one year later. However, executive function was not an explanatory mechanism for the relationship between conflict and child agency.

## 4. Discussion

This study sought to answer three research questions: (1) are parenting behaviors (i.e., reciprocity, conflict, and cooperation) and executive function bidirectionally related for preschool children with identified developmental concerns?; (2) are executive function and social–emotional skills bidirectionally related for preschool children with identified developmental concerns?; and (3) is executive function an explanatory mechanism for the relationship between parenting behaviors and social–emotional growth for children with identified developmental concerns longitudinally during the preschool years? While some work has demonstrated an association between higher overall positive and lower negative parenting behaviors and better executive function [57,58], this study is unique in its focus on examining the impact of specific dyadic parenting behaviors to disentangle their differential effects on children’s executive function.

Regarding Hypothesis 1, while parenting behaviors and executive function were related over time, there were no bidirectional effects for any specific parenting behaviors. Multiple regression analyses indicated that more conflict behaviors demonstrated by parents during the fall predicted more problems with executive functioning for children with developmental concerns during the spring of the first year of preschool. Conflict behaviors displayed by the parent, such as arguing with the child and engaging in mutual negative affect, comprise the negative parenting behaviors examined in this study. These conflict behaviors differ from other specific negative parenting behaviors (e.g., intrusiveness and detachment) previously examined in relation to executive function. This differential focus may explain why conflict behaviors were predictive of executive function in this study, while recent studies examining other aspects of negative parenting have found no association between negative parenting behaviors and children’s executive function [59,60]. The relationship between conflict behaviors and executive function suggests that programs that focus on the reduction of conflict behaviors displayed by the parent may aid in the development of children’s later executive function. This sort of intervention may be especially important for children who are doubly disadvantaged by income and developmental concern status, as improving executive function can promote resilience by setting children up on trajectories for future success [44,61].

Poorer executive function during the fall predicted less reciprocity and cooperation displayed by the parents during the spring of the first year of preschool. These findings echo other studies that demonstrate that higher executive function is predictive of greater positive parenting behaviors [62,63], while also making a unique contribution to our understanding of parent’s cooperative and reciprocal behaviors. Interventionists working with parents should be aware of the potential impact children’s executive function may have on how parents respond to and behave during interactions with their children. Mindful parenting interventions may be especially helpful for children with poorer executive function as these interventions promote greater self-awareness and regulation [64], which may in turn promote greater cooperation and reciprocity from the parent.

Regarding Hypothesis 2, findings offer support for directionality and bidirectionality for children’s specific social–emotional skills during the first year of preschool. Evidence suggests a bidirectional relationship between executive function and affection towards the parent for children with identified developmental concerns. Controlling for prior executive function in a multiple regression, displaying less agency and more affection towards the parent during the fall predicted more problems with executive function for children with identified developmental concerns during the spring of the first year of preschool. Children with less agency may be hesitant to engage with problems, express less confidence and eagerness towards the task, and be inconsistently involved across the play session [52]. These findings further support research from a previous cross-sectional study of preschool children’s agency and executive function [65] by offering support for directionality. While social–emotional skills and executive function were bidirectionally related, without an experimental design causality cannot be determined. Future research should examine if improvements in one competency will lead to improvement in both competencies or if there are additional factors that explain this association.

While the direction for the relationship between more affection towards the parent predicting greater problems with executive function was not as hypothesized, further examination of the construct offers a possible explanation. Affection towards the parent, as observationally measured for this study, was conceptualized as “looking at the parent, making eye contact and smiling, positive verbal exchanges, and other “approach” behaviors” [52] (p. 8). In this sample, affection towards the parent in the fall was rated close to a 3 on average (M = 2.77). This type of expression of affection is characterized by short, repeated bursts of affection, which are not sustained for more than a moment of time [52]. There was also no evaluation of the appropriateness of these overtures during the play interaction. Trouble with regulatory aspects of executive function may partially drive these brief positive expressions [66]. Children may receive positive reinforcement for this lack of appropriately inhibited displays by parents’ response to expressed affection. While these analyses do not allow us to make causal statements, greater affection towards the parent was related to greater reciprocity and cooperative behaviors by the parent during the fall (r = 0.68, *p* < 0.001; r = 0.35, *p* < 0.001).

In contrast, fewer concerns regarding executive function during the fall of the first year of preschool was predictive of more affection towards the parent in the spring. These findings build on previous studies that demonstrate a positive association between executive function and social–emotional skills [67,68] through greater specificity of social–emotional skills examined, especially in relation to interactions with parents. Future research should study the bidirectional relationship between affection towards the parent and executive function for children with developmental concerns in the same model. Future work could also attempt to untangle appropriately versus inappropriately expressed positive emotion through a more refined scale for evaluating children’s social–emotional skills.

The third research question was to examine executive function as an explanatory mechanism for the predictive relationship between parenting behaviors and social–emotional development for children with identified developmental concerns longitudinally across two years of preschool. No support was found for Hypothesis 3. However, a cross-lagged panel model demonstrated that conflict behaviors in the fall of the first year of preschool have an indirect effect on child outcomes across two years of preschool. More conflict behaviors in the fall predicted more conflict behaviors in the spring of the first year of preschool, which in turn predicted less child agency and poorer executive function a year later. Conflict behaviors in the fall of the first year of preschool also had an indirect effect on child executive function in the spring of the second year of preschool through executive function in the intervening spring. More conflict behaviors predicted poorer executive function.

Conflict behaviors by the parent may be an important predictor of child outcomes across the preschool years. For children who are already at risk due to low income and identified developmental concerns, the negative effects of conflict behaviors may be especially pronounced due to additional strain on children’s emotional and mental reserves [69]. Overall, parents in this study displayed low levels of conflict behaviors, with 84.3% of parents in the fall and 89.2% of parents in the spring of the first year displaying very low levels of conflict during the observed play interaction. Families with parents who do engage in higher levels of conflict may differ from low-conflict parent–child dyads in ways that may inform the development of children’s executive function and social–emotional skills. Future research should more closely examine theories of additive risk [69] in relation to parent–child conflict. Intervention research should focus on factors that may predict higher parental conflict behaviors and develop programs to reduce these parenting behaviors. Future research should examine these processes longitudinally beyond preschool to enable a more comprehensive understanding of how parent conflict behaviors, executive function, and social–emotional competencies impact children’s long-term success.

In addition, future research should examine the impact of parent conflict behaviors in the parent–child dyad in the context of stress. As this study did not include parental stress as a predictor or covariate in the model, parents displaying higher levels of conflict behaviors may be an indicator of greater stress. In a diverse sample of low-income families with children attending publicly funded preschools, higher parenting stress was predictive of greater parent–child conflict [70]. Similarly, parents experiencing higher levels of stress about their marital relationship [71] and housing insecurity [66] were more likely to exhibit conflict behaviors when interacting with their children. As higher levels of parenting stress are predictive of poorer child self-regulation and executive function [72], stress could have a significant contribution in a reexamination of these models. Parental education is also negatively associated with parent–child conflict behaviors [73]. Future research should examine the role of potential intervening variables such as parenting stress, parent education, and housing insecurity in disrupting the hypothesized relationship between conflict, executive function, and social–emotional competencies.

This study has several limitations. First, executive function was only measured using teacher report on the BRIEF-P [49]. Multiple assessments of executive function may better measure the underlying unitary structure of executive function hypothesized to exist in preschool [37]. Although we used the unidimensional factor structure suggested by Spiegel and colleagues [50], multiple more robust measures of executive function would improve the rigor of this study. Second, parenting is a complex construction comprised of multiple dimensions including parental sensitivity, discipline, communication, emotional support, etc. As such, the examination of dyadic parenting behaviors such as parental conflict behaviors offers a limited perspective on parenting. Third, as previously discussed, a multidimensional observational measure may have allowed for a more refined examination of certain social–emotional skills. For example, future work could examine affection towards the parent using interval coding to closely monitor the duration of behaviors or an adapted scheme that allows for the assessment of the quality and appropriateness of the behavior. However, future researchers would need to weigh the time needed to learn a more complicated coding scheme against the ability of the current well-validated coding system to answer questions of interest. Fourth, Hypothesis 3 was examined using a cross-lagged panel model that only allows for the examination of change over time compared with change at the individual level. Fifth, while the current study examines these processes in low-income families, contextual factors such as educational resources or economic stress are not explored. Future research should address how these variables interact with dyadic parenting behaviors and child development.

Despite these limitations, this study is an important contribution to our understanding of the associations between dyadic parenting behaviors, executive function, and social–emotional skills. This study examined these processes in a unique and understudied sample by focusing on children with identified developmental concerns who may be at particular risk for underdeveloped executive function or social–emotional skills [63]. Further, observationally assessing specific parenting behaviors and children’s social–emotional skills allowed this study to tease apart previous relations found between broad constructs of parenting, executive function, and social–emotional development.

## 5. Conclusions

This study examines the complexity of change in behaviors over time. For example, parenting behaviors did not always predict later parenting behaviors across the two years of preschool. Further, parenting behaviors and children’s executive function in the fall of the first year of preschool did not directly predict child outcomes two years later. As executive function was not an explanatory mechanism for the relationship between parenting and children’s social–emotional skills, these findings point to the importance of identifying other mechanisms that may disrupt predictive influences on children’s development. Results also demonstrate the robust impact of conflict displayed by parents during interactions with their child on children’s development across two years of preschool. Higher levels of conflict in the fall predicted parents displaying more conflict behaviors in the spring of the child’s first year of preschool. Higher levels of conflict in the spring then predicted children having poorer executive function and less agency a year later. This suggests the importance of intervening to reduce parent–child conflict in early childhood to promote children’s long-term cognitive and social–emotional functioning, particularly for children with identified concerns upon entry into publicly funded preschool programs.

## Figures and Tables

**Table 1:** Family demographic characteristics during the fall of the child’s first year of preschool.

	Child	Parent
Mean Age	46.02 months (*SD* = 3.67) (range = 39–54)	29.6 years (*SD* = 5.9) (range = 19–49)
Gender		
Male	56.1%	12.8%
Female	43.9%	87.2%
Race		
White	70.5%	80.3%
Black	4.1%	3.4%
American Indian/Native Alaskan	1.6%	3.4%
Asian	0.4%	0.8%
Two or more races	12.3%	3.4%
Other	11.1%	8.8%
Ethnicity		
Latino/Hispanic	30.1%	23.7%
Home Language		
Spanish	19.0%	17.6%
English	81.0%	82.4%
Individualized Education Program	29.8%	
Marital Status		
Single		32.0%
Partnered		68.0%
Highest Level of Education		
<High school diploma		23.0%
High school diploma/GED		28.5%
Some training beyond HS/no degree		25.5%
Two-year degree		12.8%
Four-year or more degree		10.2%

**Table 2:** Descriptive statistics for parenting behaviors, executive function, and social–emotional skills across two years of preschool.

	Time 1 Fall Year 1 Mean (*SD*) N ^a^ = 230	Time 2 Spring Year 1 Mean (*SD*) N ^a^ = 202	Time 3 Spring Year 2 Mean (*SD*) N ^a^ = 109
Parenting Behaviors ^b^			
Reciprocity	2.69 (0.70)	2.63 (0.65)	2.72 (0.67)
Conflict	1.17 (0.42)	1.11 (0.33)	1.06 (0.23)
Cooperation	2.52 (0.64)	2.63 (0.63)	2.77 (0.68)
Concerns Regarding Child Executive Function ^c^			
Executive Function	58.22 (14.23)	57.49 (12.24)	50.72 (11.75)
Child Social–Emotional Skills ^d^			
Agency	4.06 (0.77)	4.22 (0.70)	4.28 (0.64)
Compliance	4.30 (0.81)	4.14 (0.79)	4.01 (0.78)
Affection towards the Parent	2.77 (0.76)	2.61 (0.77)	2.55 (0.76)
Avoidance of the Parent	1.37 (0.72)	1.41 (0.69)	1.35 (0.63)
Negativity	1.66 (0.76)	1.62 (0.78)	1.61 (0.77)

^a^ Sample size of coded parent–child video-taped interactions. ^b^ Scores range from 1 to 5. ^c^ Scores are converted to standard scores based on the child’s age; M = 50, SD = 10. Higher scores denote more concerns, or poorer executive function. ^d^ Scores range from 1 to 5.

**Table 3:** Regression analysis for parenting behaviors in the fall predicting child executive function during the spring of the first year of preschool.

		B	SE B	β	F	R^2^	ES
	Executive Function				109.29 ***	0.73	2.70
*Parent*	Reciprocity	−0.19	0.80	−0.01			
	Conflict	3.07	1.22	0.10 *			
	Cooperation	−0.29	0.29	−0.32			
	Child Executive Function ^a^	0.59	0.06	0.71 ***			

Note. Treatment condition is controlled for in all analyses. ^a^ Higher scores indicate greater teacher-rated concern regarding the child’s executive function. * *p* < 0.05; *** *p* < 0.001.

**Table 4:** Regression analysis for child executive function in the fall predicting parenting behaviors during the spring of the first year of preschool.

	B	SE B	β	F	R^2^	ES
Reciprocity				3.72 **	0.06	0.06
Child Executive Function ^a^	−0.01	0.00	−0.14 *			
Reciprocity	0.16	0.06	0.17 *			
Conflict				1.45	0.02	0.02
Child Executive Function	0.00	0.00	0.05			
Conflict	0.12	0.06	0.14			
Cooperation				3.39 **	0.05	0.05
Child Executive Function	−0.01	0.00	−0.14 *			
Cooperation	0.17	0.07	0.17			

Note. Treatment condition is controlled for in all analyses. ^a^ Higher scores indicate greater teacher-rated concern regarding the child’s executive function. ** For test statistic: *p* < 0.02, statistically significant with a Bonferroni correction; * for predictors: *p* < 0.05.

**Table 5:** Regression analysis for executive function during fall predicting child social–emotional skills during the spring of the first year of preschool.

	B	SE B	β	F	R^2^	ES
Agency				2.45	0.04	0.04
Child Executive Function ^a^	0.00	0.00	−0.08			
Agency	0.15	0.07	0.17			
Compliance				10.15 ****	0.14	0.16
Child Executive Function	0.00	0.00	−0.07			
Compliance	0.35	0.07	0.36 ***			
Avoidance				7.95 ****	0.11	0.12
Child Executive Function	0.00	0.00	0.06			
Avoidance	0.31	0.07	0.32 ***			
Affection Towards the Parent				4.89 ****	0.07	0.08
Child Executive Function	−0.01	0.00	−0.18 *			
Affection	0.21	0.07	0.21 **			
Negativity				7.77 ****	0.11	0.12
Child Executive Function	0.00	0.00	−0.01			
Negativity	0.35	0.07	0.34 ***			

Note. Treatment condition is controlled for in all analyses. ^a^ Higher scores indicate greater teacher-rated concern regarding the child’s executive function. **** For test statistic: *p* < 0.01, statistically significant with a Bonferroni correction; * for predictors: *p* < 0.05; ** for predictors: *p* < 0.01; *** for predictors: *p* < 0.001.

**Table 6:** Regression analysis for child social–emotional development during fall predicting child executive function during the spring of the first year of preschool.

	B	SE B	β	F	R^2^	ES
Executive Function ^a^				77.86 ***	0.74	2.85
Agency	−1.53	0.78	−0.08 *			
Compliance	0.04	0.94	0.00			
Avoidance	−0.49	0.94	−0.03			
Affection	1.20	0.71	0.07 *			
Negativity	1.07	0.87	0.06			
Child Executive Function	0.82	0.04	0.83 ***			

Note. Treatment condition is controlled for in all analyses. ^a^ Higher scores indicate greater teacher-rated concern regarding the child’s executive function. * *p* < 0.05; *** *p* < 0.001.

## Data Availability

Please contact the corresponding author for inquiries about access to the data. Data are still being archived.
